# Ultra-high spatial resolution at photon-counting computed tomography: technical insights and sustainable applications in cardiothoracic imaging

**DOI:** 10.1186/s41747-025-00656-0

**Published:** 2026-01-05

**Authors:** Mariaelena Occhipinti, Alberto Clemente, Carmelo De Gori, Vincenzo Positano, Danilo Neglia, Simona Celi, Antonella Meloni, Sergio Berti, Filippo Cademartiri

**Affiliations:** 1 Department of Imaging, Fondazione Toscana Gabriele Monasterio/CNR, S. Cataldo Hospital, Via G. Moruzzi, Pisa, Italy; 2 Bioengineering Department, Fondazione Toscana Gabriele Monasterio/CNR, Via G. Moruzzi, Pisa, Italy; 3 Cardiology Department, Fondazione Toscana Gabriele Monasterio/CNR, S. Cataldo Hospital, Via G. Moruzzi, Pisa, Italy; 4 Department of BioEngineering, Fondazione Toscana Gabriele Monasterio/CNR, S. Cataldo Hospital, Via G. Moruzzi, Pisa, Italy; 5 Department of Interventional Cardiology, Fondazione Toscana Gabriele Monasterio/CNR, S. Cataldo Hospital, Via G. Moruzzi, Pisa, Italy; 6 Radiology, IRCCS SYNLAB SDN, Naples, Italy

**Keywords:** Cardiac, CdZnTe detector, Chest, Coronary artery disease, Multidetector computed tomography

## Abstract

**Abstract:**

The latest technological advancements in CT enable the exploration of unprecedented limits of spatial resolution in *in vivo* imaging. Nowadays, ultra-high-resolution imaging is available by using CT with detector elements at or smaller than 0.25 mm along the *z*-axis, like those used on photon-counting CT (PCCT) scanners. However, spatial resolution represents a complex criterion of imaging performance affected not only by detector elements, but also by other complex variables that can interact with each other. Knowledge of these variables and the metrics to evaluate spatial resolution is key to performing accurate cardiothoracic examinations with optimized CT protocols, which can eventually reduce acquisition times and radiation doses. This opens to a sustainable cardiothoracic radiology that permits accurate cardiac CT evaluations also in patients previously excluded, due to high calcium score, metallic stents or obesity, and allows to reduce radiation doses to never-seen levels. In this article, we review the technical advancements that allowed such an increase in spatial resolution in PCCT, along with all technical determinants of spatial resolution, the metrics to evaluate it, the clinical impact of UHR at PCCT and its challenges on cardiothoracic imaging.

**Relevance statement:**

Knowledge of the ultra-high spatial resolution capabilities of new photon-counting CT technology is key to its best uses — performing accurate diagnostic examinations at unmatched low radiation doses and scanning patients previously excluded from cardiac CT examinations.

**Key Points:**

- Photon-counting CT scanners enable radiologists to evaluate cardiothoracic examinations with an exceptional spatial resolution, along with spectral information and dose reduction.
- The ultra-high spatial resolution in cardiovascular imaging enables accurate assessment in patients previously excluded, such as obese, those with extensive calcifications or metallic stents.
- Ultra-high spatial resolution empowers the visualization and follow-up of focal and diffuse lung diseases at unmatched accuracy and low radiation doses.

**Graphical Abstract:**

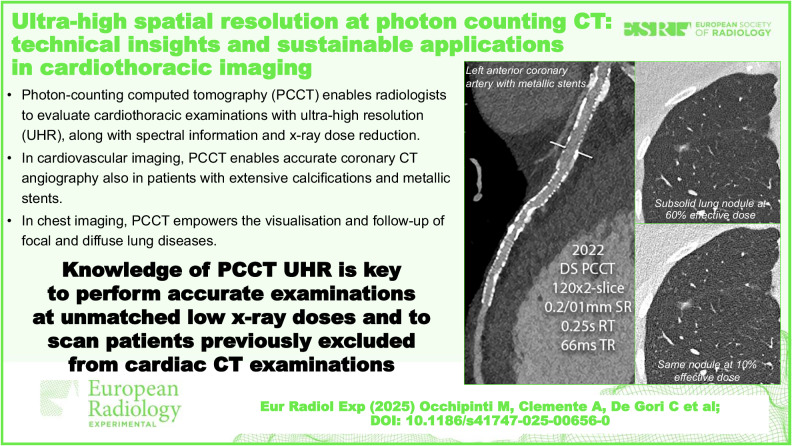

## Introduction

Spatial resolution is the ability to differentiate two points or adjacent structures as distinct from each other in a diagnostic image. It is an important criterion to measure the imaging performance of an imaging modality and of a specific imaging system. This technical characteristic is of utmost importance when the focus is on small anatomy and pathologic structures, such as coronaries, cardiac valves, small airways, lung vasculature and interstitium [1, 2]. Insufficient spatial resolution may create partial volume artifacts, thus hampering the diagnostic capabilities in cardiothoracic imaging [3].

Among cross-sectional diagnostic modalities, CT provides the highest spatial resolution, as compared to magnetic resonance (MR) and nuclear medicine (PET and SPECT) scanners, as well as transthoracic ultrasounds. Over time, the performance of CT has significantly and continuously improved across several generations of scanners. Recently, the introduction of the latest CT scanners, which rely on the direct characterization and count of photons (*i.e*., photon-counting CT, PCCT), enabled further improvements as compared to previous conventional EID (energy-integrating detector) CT scanners. Amongst all improvements, spatial resolution represents the most important and immediately available one.

Nowadays, PCCT scanners allow ultra-high resolution (UHR) *in vivo* imaging, with the use of detector elements at or smaller than 0.25 mm along the *z*-axis, according to the definition by the international expert consensus on standardized medical terminology for cardiac CT [4]. UHR on PCCT scanners led to an increase in spatial resolution up to 0.11 mm [5, 6]. To date, only medical micro-CT and nano-CT scanners can achieve a superior spatial resolution (about 0.005 mm and 0.1 micron, respectively), but only in *ex vivo* imaging and in preclinical settings [6, 7].

In this article, we review the technical advancements that allowed UHR imaging in the last generation of clinical CT scanners, the metrics to evaluate spatial resolution, the clinical impact of UHR at PCCT and its current challenges with a particular focus on cardiothoracic imaging.

## Determinants of spatial resolution

Spatial resolution on CT depends on several factors, either inherent to the CT scanner type (thus unmodifiable) or modifiable during the image acquisition or reconstruction phase. They include detector cell size, X-ray tube factors, projection sampling frequency, acquisition and reconstruction geometry (FOV, pitch, matrix size), and reconstruction algorithms [6, 8, 9].

Although interlinked, it is fundamental to differentiate between the spatial resolution of the CT scanner and of the reconstructed image. X-ray tube focal spot size, number of projections per rotation and detector cell size along the fan direction determine the maximum in-plane spatial resolution of the scanner, while the detector cell size in the row direction determines the minimum slice width.

### Detector cell size

The decrease in size of the detector element is one of the most effective methods to increase spatial resolution.

**Fig. 1:**
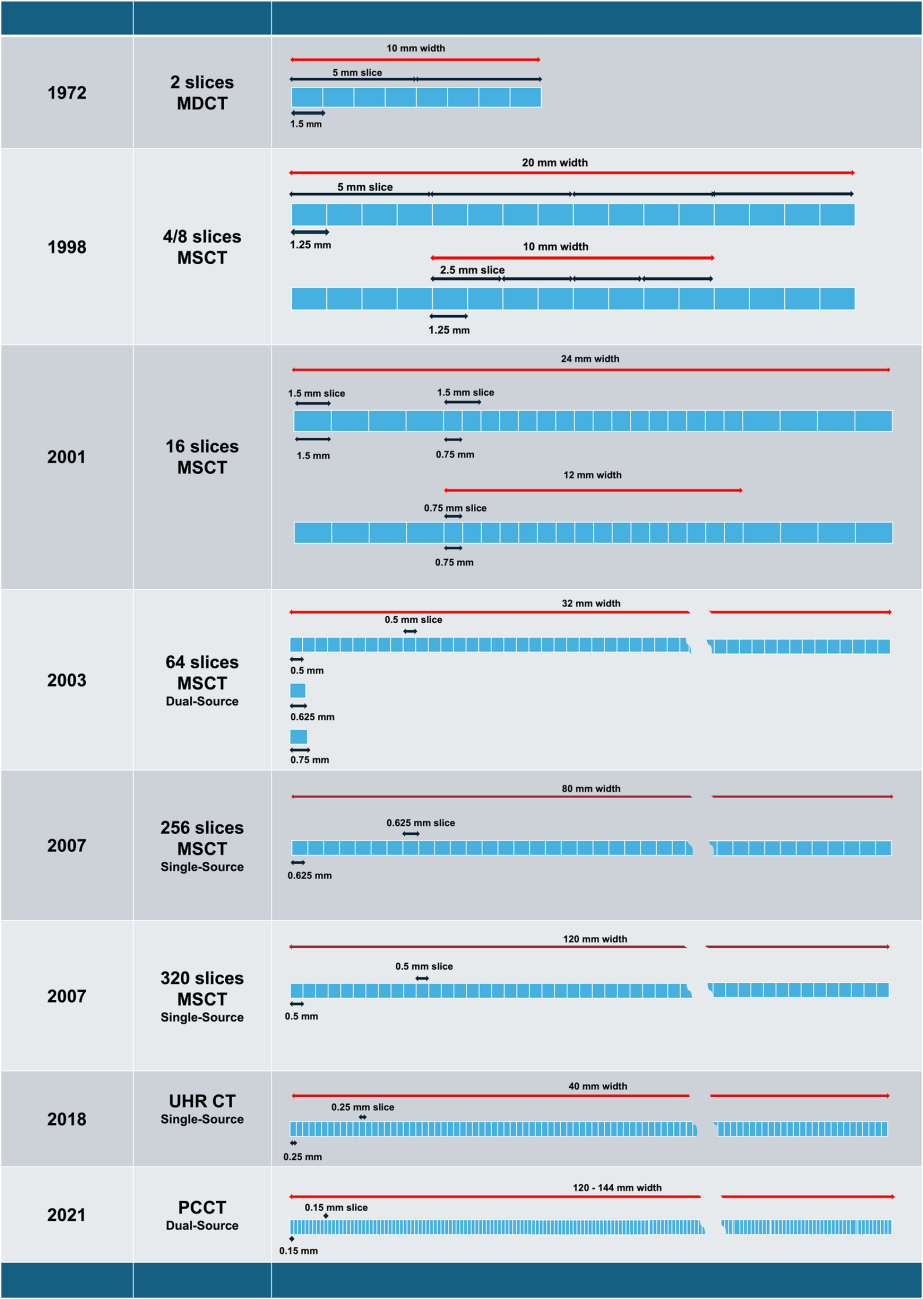
Evolution of computed tomography (CT) detectors from energy-integrating detectors (EIDs) to last-generation PCDs. First and early multidetector CT (2–4 slices) architectures were based on 1.25 mm detectors linked in 16-element groups to obtain 5 mm and 2.5 mm slice thickness depending on beam width. The architecture also allowed the acquisition of 8 slices (2.5 or 1.25 thickness). 16-slice multidetector CT introduced an uneven distribution of detector sizes over array width, allowing the acquisition of 1.5/0.75 mm slices (Siemens/Philips architecture shown). 64-slice multidetector CT further reduced detector size (down to 0.5 mm, Toshiba architecture shown, or 0.625 mm or 0.75 mm), increasing the array size [79, 80]. In ultra-high resolution (UHR) CT (Canon Aquilion Precision), the detector size was further reduced to 0.25 × 0.25. Finally, PCCT introduced a new class of detectors of about 0.15 mm in size. EID, Energy-integrating detector; PCCT, Photon-counting CT; UHR, Ultra-high resolution

For more than a decade, the physical dimensions of clinical detectors of conventional multidetector CT scanners remained unchanged at 0.5–0.625 mm at the isocenter of the scanner along the *z*-axis and approximately 0.3 mm in the *x*- to *y*-axes. However, the number of detector rows gradually increased from 2- to 640-slice CT scanners (Fig. 1), allowing a parallel increase in spatial resolution from 1 up to 0.17 mm (Table 1) [10].

**Table 1:** Spatial resolutions of PCCT systems used in clinical practice at different acquisition modes

Vendor	PCD material	PCD element size (mm^2^)	PCD size per resolution mode	z-coverage	Matrix sizes available	Spatial resolution (in-plane/through-plane) (mm) OR TTF/MTF
Siemens Healthineers	Cadmium telluride	Detector elements: 0.225 × 0.225 [73]				
			Standard/Macro mode: 0.9 × 0.9 mm (4 × 4; 0.5 mm at isocenter) [73, 74]	N.R.	512	0.24/N.R.
			HR/Sharp mode: 0.45 × 0.45 mm (2 × 2; 0.25 at isocenter) [73, 74]	2 × 144 × 0.4 mm (57.6 mm)	512, 768, 1,024	N.R.
			UHR mode: 0.151 × 0.176 mm [4]	2 × 120 × 0.2 mm (24 mm)	512, 768, 1,024	0.11/0.16 [14]
GE Healthcare	Silicon (Prismatic Sensors)	0.5 × 0.4	UHR mode	N.R.	N.R.	14 to 28 lp/mm on phantoms [75]
Philips Healthcare	Cadmium zinc telluride	0.27 × 0.27 [76]	UHR mode	64 × 0.3 mm (17.5 mm)	512, 1,024, 2,048	- TTF_10_ ~30% better than EID-CT counterpart - TTF_50_ ~35% better than EID-CT counterpart
Canon	Cadmium zinc telluride	0.25 × 0.25 (1,792 channels per 160 rows) [77]	Normal resolution mode: 0.50 × 0.50 mm	40 mm	512	N.R.
			HR mode: 0.25 × 0.50 mm		512, 1,024, 2,048	N.R.
			Super HR mode: 0.25 × 0.25 mm		512, 1,024, 2,048	MTF_10_ 41 lp/cm

*HR* High resolution, *MTF* Modulation transfer function, *N.R.* Not reported, *TTF* Target transfer function, *UHR* Ultra-high resolution

A step forward in spatial resolution was then accomplished by the advent of UHR-CT imaging, *i.e.*, CT with detector elements at or smaller than 0.25 mm along the *z*-axis, as defined based on hardware characteristics by the international expert consensus on standardized medical terminology for cardiac CT [4]. In 2018 a CT scanner with detectors 2.5 mm thick and a maximum resolution of 0.15 mm (50 lp/cm) (Aquilion Precision CT system, Canon) enabled the first UHR imaging [11]. The detectors used in this first UHR system were EIDs, like the ones used in previous CT scanners. EIDs can convert interacting photons into visible light and subsequently into electrical signals. However, the visible light generated can then create a cross-talk between neighboring elements of the photodiode. The application of septa and comb filters tried to overcome this issue, at a cost of dose efficiency (as they represent a dead space) and spatial resolution.

A further advance in UHR imaging arrived with the premarket FDA clearance of the first PCCT scanner in 2021 (NAEOTOM Alpha, Siemens). PCCT relies on photon-counting detectors (PCD) constituted by a semiconductor (in Cd-Te, Cd-Zn-Te or silicon crystals) that directly converts the X-ray photons into digital electric signals, abolishing conversion time and the need for a scintillator and septa [12, 13]. The lack of septa optimizes geometric dose efficiency and allows smaller detector sizes [6]. This turns into increasing spatial resolution, which arrives up to 0.11 mm in-plane and 0.16 mm cross-plane [14] (see Table 1).

**Fig. 2:**
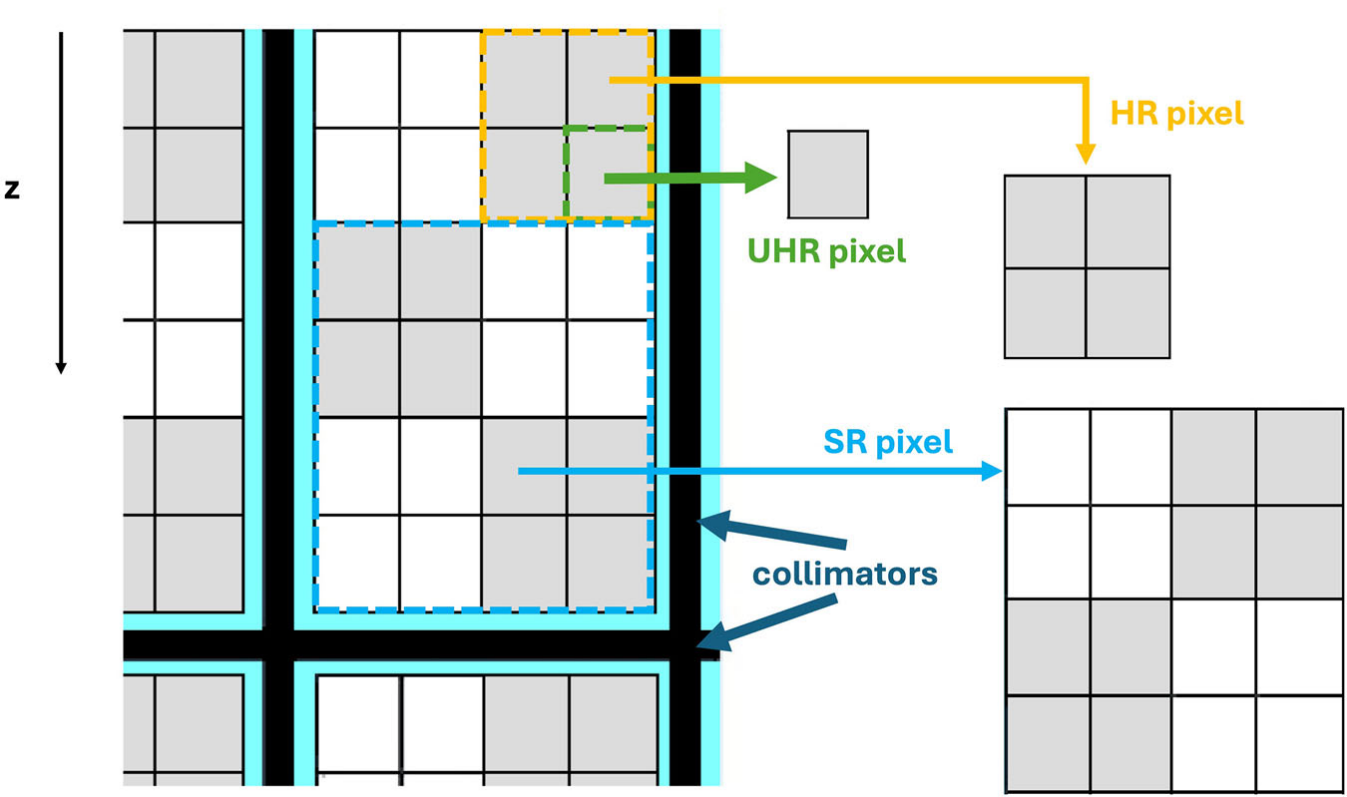
Layout and acquisition modes in PCCT. Three layouts of detectors can be used with clinically available PCCT scanners: SR (standard resolution), HR (high resolution), UHR (ultra-high resolution). Photon-counting detectors (PCDs) arrangement in a PCCT commercial scanner. Subpixels can be arranged in 2 × 2 (HR) or 4 × 4 (SR) blocks to improve signal-to-noise ratio and reduce radiation dose, with a parallel loss in image resolution as compared to UHR mode

In PCDs, individual photons can be counted and registered with respect to energy, under ideal conditions. This allows higher spatial resolution and inherent spectral imaging, which translate into a higher signal-to-noise ratio (SNR). PCDs are arranged in subpixel blocks separated by collimators to reduce scattered radiation. Then the detector pixel size can be arranged in different ways according to the desired resolution mode (see Fig. 2, Table 1): standard (SR), high resolution (HR), and ultra-high resolution (UHR).

In UHR mode, the resolution of each subpixel (0.150 × 0.176 mm in size measured at isocenter) is fully exploited [15], bringing a remarkable spatial resolution of 0.11 mm (in-plane) by using a slice thickness of 0.2–0.25 mm, slice increment of 0.1 mm, matrix 512 × 512, 768 × 768, 1,024 × 1,024, or 2,048 × 2,048. This mode enables HR images in both in-plane and through-plane to evaluate fine lesions and anatomical structures (*e.g*., lung interstitium, small coronary plaques, in-stent re-stenosis).

If UHR is not strictly needed, the radiation dose can be reduced by using HR mode. In HR mode, subpixels are grouped in 2 × 2 blocks, leading to a 2 × 3 array. The size of each block doubles the size of subpixels and section thickness with respect to the UHR mode. HR mode works with a slice thickness of 0.4–0.5 mm, matrix size 512 × 512, 768 × 768, 1,024 × 1,024, or 2,048 × 2,048, while retaining a temporal resolution of 66 ms in hardware and maintaining full dose efficiency.

Early prototypes of PCCT scanners implemented a standard resolution (SR) mode, where 4 × 4 subpixels are binned in “MACRO pixels” with a size of 0.9 × 0.9 mm^2^ (0.5 × 0.2 mm^2^ at the isocenter) comparable to conventional CT systems [16]. SR mode works with a slice thickness of 0.4–0.5 mm, matrix size 512 × 512, and an in-plane resolution of 0.24 mm.

HR and UHR modes have a 70% and 87% improvement in the in-plane spatial resolution, respectively, compared with the SR mode and with HR images elaborated by EID-based CT [17].

All three modes have at least 2 energy thresholds, which enable single-source, single-kV, and simultaneous dual-energy CT. The spectral separation enabled by PCDs is theoretically more effective than the spectral CT performed using EIDs, due not only to the capability of PCDs to count and register individual photons using adjustable energy thresholds but also to the lack of electronic noise and of dead space between detectors, and to the improved energy weighting of the low-energy photons and the improved spatial resolution [18]. PCCT can use more than two energy bins, improving the ability to quantify materials within a given space and making multi-contrast imaging possible [6]. However, this PCCT scanner ability still needs to be investigated in clinical centers.

### X-ray tube factors

X-ray tube factors (*i.e*., focal spot size, position of the tube and gantry rotation time) are unmodifiable factors that can affect spatial resolution, and they are in-built within the CT scanner. The smaller the X-ray focal spot size, the smaller the blur in the image. In UHR-CT imaging, the focal spot has been reduced to a width of 0.4 mm and a length of 0.5 mm, compared to the 0.9 mm width and 0.8 mm length in conventional EID-CT. Noteworthy, focal spot size may vary at different tube voltages [19]. As UHR imaging is performed at high current values, focal spot blooming due to high temperature can partially counteract the benefits of the small focal spot size. Focal spot multiplexing technique, allowed by the digital nature of PCCT, could potentially mitigate this issue by using multiple focal tracks and rapid switching [20].

Gantry rotation time and extent (*i.e*., half *versus* full scan) may also affect spatial resolution in motion-sensitive applications, such as cardiac CT. Faster gantry rotation, while reducing scan time, can further increase the image noise present in UHR images. Hence, rotation time and extent may have a considerable influence on image quality in UHR-PCCT and should be adapted to the specific anatomical target [21].

### Projection sampling

The number of projections per rotation is a factor affecting the spatial resolution away from the isocenter toward the outer parts of a large FOV (*e.g*., lung). The projection sampling frequency can realize values of up to 8 kHz and cannot be influenced by the user, as it is tied to the selected acquisition scan mode (*e.g*., rotation time).

### Acquisition and reconstruction geometry (field of view -FOV, pitch, and matrix size)

The combination of FOV and matrix size determines the maximum theoretical resolution, which can be displayed in an image and therefore needs to fit to the applied reconstruction kernel and its predefined resolution characteristics. Smaller reconstruction FOV (*i.e*., 5 × 5 cm) at a fixed matrix size can be very helpful in the visualization of details in the coronary tree and in the most peripheral airways in case of a sharp reconstruction kernel.

The effect of different pitch values on in-plane and through-plane spatial resolution in PCCT in cardiothoracic imaging studies has to be exploited. Euler et al tested the effect of 4 pitch values by using a single-source wide-coverage fast-kV-switching dual-energy computed tomography (DECT), finding a non-significant difference in the mean spatial frequency in phantoms (see “Performance metrics” below) [22].

Matrix size influences the spatial resolution of a reconstructed image as a limiting factor if it is insufficient for the selected reconstruction kernel. The improved spatial resolution of the scanner realized in the form of sharp kernel reconstructions therefore mandates access to larger matrix sizes of 768 × 768 or 1,024 × 1,024 in a typical FOV above 200 mm [8]. By using these large matrices, the pixel size decreases accordingly. In a chest CT scan with a FOV of 32 cm, the theoretical pixel size is 0.625 mm for 512 × 512, 0.313 mm for 1,024 × 1,024, 0.156 mm for 2,048 × 2,048.

Higher matrix sizes enable increased image sharpness in the case of a large FOV combined with a sharp reconstruction kernel at the expense of higher noise. Noise increases in parallel with matrix size (average SD values of air equal to 6.32 HU for 512 × 512, 22.42 HU for 1,024 × 1,024, 33.72 HU for 2,048 × 2,048) [11], on average 16% higher for 1,024 × 1,024 compared to 512 × 512 matrix [23]. Contrariwise, streak artifacts do not change significantly across matrix sizes [11].

The matrix size defines the maximum spatial resolution of the image, and this maximum spatial resolution (0.11–0.15 mm) should always exceed the kernel-defined spatial resolution of the CT scanner [11]. In a reconstruction with a given spatial resolution, the spatial resolution of the reconstructed image should therefore be optimized by choosing the correct match between FOV and matrix size, which is often provided automatically by a dedicated automatic functionality on scanner.

Some studies have assessed the effect of 1,024-pixel matrix in UHR-CT using both EID and PCCT systems [11, 23–26]. Bartlett et al [26] demonstrated an increased ability of the radiologist to visualize higher-order airways and their walls by using 1,024-pixel matrix with B46 or Q65 reconstruction kernels at PCCT, as compared to 1,024-pixel matrix with B46 at EID-CT.

### Reconstruction algorithms

To take advantage of the higher resolution data in PCCT than in EID-CT, sharp kernels are needed. However, the use of sharp kernels increases the image noise, which may then be mitigated by denoising methods and slice thickness increase [27, 28]. On PCCT, the kernel should be accurately chosen according to the type of examination and the acquisition mode chosen.

**Fig. 3:**
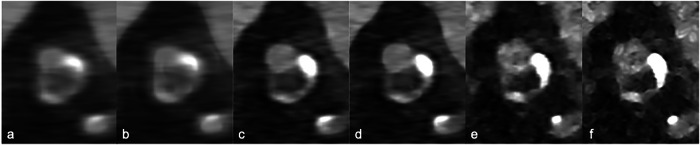
Example of cardiac computed tomography angiography (CCTA) imaging with PCCT using different matrix sizes and reconstruction kernels. Axial CCTA images of a left anterior descending artery, showing a partly calcified plaque with positive remodeling (Dual Source PCCT Naeotom Alpha, Siemens, 144 × 0.2 mm × 2 sources, temporal resolution 66 ms, Heart-Rate: 55 bpm, R-R temporal phase: -180 ms, recon FOV 5 × 5 cm, voxel size 0.1 mm^3^, window level 1,400 W/400 C). Reconstruction kernels: Bv36 (**a**, **b**), Bv48 (**c**, **d**), Bv72 (**e**, **f**). Matrix sizes: 512 × 512 (**a**, **c**, **e**), 1,024 × 1,024 (**b**, **d**, **f**). As the kernel strength and the matrix size increase, the characterization of plaques with calcified components improves. CCTA, Cardiac CT angiography; PCCT, Photon-counting CT; UHR, Ultra-high resolution; FOV, Field of view; bpm, Beats per minute

In cardiovascular studies, medium-soft kernels are commonly used in clinical routine. However, sharper kernels can reduce calcium blooming, identify significant stenosis and in-stent re-stenosis, and reproduce invasive FFR more faithfully than smoother ones (Bv49 *versus* Bv40, respectively) [29, 30]. Rajagopal et al [27] found that PCCT modes contained more high-frequency signal in their initial acquisitions that became more apparent with sharper kernels (B46f *versus* B30f). This is particularly important for the accurate evaluation of coronary plaques (see Fig. 3) and their stenosis. PCCT enables a more accurate and less variable stenosis quantification and categorization as compared with standard resolution CCTA in patients with a high coronary calcium load, also thanks to the use of sharper kernels (Bv64 in UHR *versus* Bv40 in standard reconstructions) [31].

**Fig. 4:**
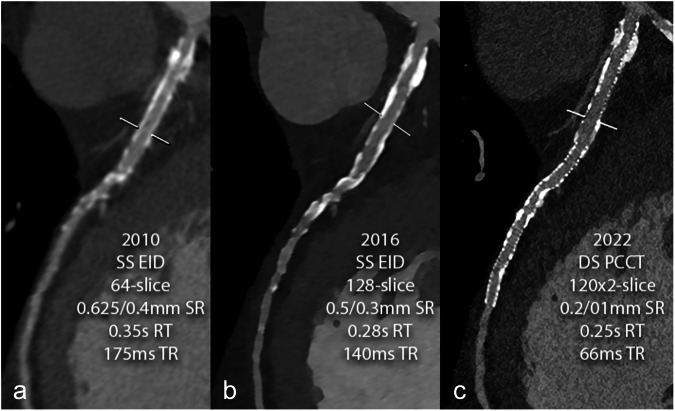
Clinical evolution of cardiac CT angiography (CCTA) performance across 15 years in the assessment of stent patency. The same approximately 70-year-old patient with known coronary artery disease, who was followed for a long time using CCTA for multiple coronary stents; the image focuses on the left anterior coronary artery with several stents from the ostium; it is evident that, along the follow-up, more stents have been positioned more and more distally along the vessel. **a** CCTA scan performed in 2010 with a single-source (SS) 64-slice CT with 0.625 mm slice thickness and 175 ms temporal resolution, reconstructed with a curved multiplanar reconstruction (MPR). **b** The same curved MPR is derived from a CT scan performed in 2016 with a SS 128-slice CT with 0.5 mm slice thickness and 140 ms temporal resolution. **c** The same curved MPR is derived from a dual source (DS) PCCT scan performed in 2022 on a first-generation DS 120 × 0.2 mm × 2, with 0.2-mm slice thickness and 66 ms temporal resolution. The progression in image sharpness is evident, as well as the reduction of the impact of motion artifacts and blooming artifacts related to metal stent structs and calcifications. CCTA, Cardiac CT angiography; CAD, Coronary artery disease; DS, Dual source; EID, Energy-integrating detector; MPR, Multiplanar reconstruction; PCD, Photon-counting detector; PCCT, Photon-counting CT; RT, Rotation time of the gantry; SR, Spatial resolution; SS, Single source; TR, Temporal resolution (in hardware)

Moreover, PCCT has proved to be superior to EID-CT in the assessment of in-stent re-stenosis (Fig. 4). Vattay et al found that UHR mode with Bv56, QIR4, and 0.2 mm reconstructions is the preferred way by readers for assessing stents [32]. “Optimized” vascular kernels (like Bv72) can further reduce the central hyperdense artifact within the stent and the hypodense artifacts adjacent to the stent strut [33]. More recently, higher kernels for cardiovascular studies (*i.e*., Bv76, Bv80, Bv89) have been released, and their clinical impact has yet to be determined.

In chest imaging, last-generation kernels on PCCT increase the spatial resolution at very low expense of image noise and enable readers to better identify bronchial walls of 3^rd^ and 4^th^ order bronchi without compromising nodule evaluation [24, 26, 27, 34, 35]. For the lung study in particular, specialized kernels like Bl60 and Bl64 have been released. Milos et al have tested the performance of Bl64 as compared to Bl56 and Bl60 in combination with three different slice thicknesses (0.2, 0.4, and 1 mm) [36], finding that only the reconstruction using Bl64 at 0.4 mm thickness yielded improved bronchial division identification and bronchial wall and pulmonary fissure sharpness without any diagnostic loss in pulmonary vessel sharpness, or conspicuity of nodules, or other pathologies.

Beyond the possibilities offered by the kernel filtering to enhance image quality, novel iterative reconstruction algorithms called quantitative iterative reconstructions (QIR) have also been introduced with the advent of PCCT [37]. QIR is specifically tailored for PCCT, has four strength levels (QIR 1–4), and performs a statistical optimization of all spectral data based on local signal-to-noise analysis of the data content and then subtraction of detected noise at each iterative step [38]. The QIR algorithm enhances the image quality of CCTA datasets without compromising image sharpness or accurate measurements, with the most prominent benefits at the highest strength level (QIR4) [39]. Sartoretti et al tested QIR levels on low-dose UHR chest PCCT [40], finding that lung attenuation is highly comparable across QIR strengths, with QIR4 having better subjective noise and QIR3 being the best for image sharpness and overall image quality. Vecsey-Nagy et al tested QIR levels *in vivo* and *in vitro* on a dynamic vessel phantom containing calcified lesions scanned at different heart rates, finding that increasing QIR levels significantly reduce objective image noise and increase signal- and contrast-to-noise ratios [39].

## Performance metrics to evaluate spatial resolution

There are qualitative and quantitative ways to assess the intrinsic spatial resolution properties of the imaging system.

Qualitative assessment can be performed by readers grading the blurring of objects on the images. Ranked Likert scale is the most used and accurate qualitative approach to assess biomedical images, along with pairwise comparison [41]. Different kernels, matrices, slice thickness, as well as image quality and visibility of structures, have been tested on a 5-point Likert scale to assess the performance of PCCT *versus* EID-CT on clinical endpoints describing sharpness and noise of the image, eventually affecting spatial resolution [24, 35, 36, 42, 43].

Quantitative characterization of the spatial resolution should be performed both in-plane and cross-plane (*z*-axis) under reference conditions. There are different approaches to quantify spatial resolution, either based on distance (full width at half maximum—FWHM) or on contrast (line-pair—lp). The spatial resolution performance is most commonly measured by the highest number of lp/mm (*i.e*., the densest grid) that can be resolved in reconstructed images at a given relative contrast (*e.g*., 10% or 2% MTF—Modulation Transfer Function) [8].

If nonlinear processing (*e.g*., iterative reconstructions) is used, lp patterns may not faithfully reflect the system’s ability to resolve low-contrast features. Thus, the American Association of Physicists in Medicine (AAPM Task Group 233) recommended the use of the Task Transfer Function (TTF) [44]. The spatial frequencies at which TTF reaches 50% and 10% are denoted as f_50_ and f_10_, in the in-plane direction, and as zf_50_ and zf_10_ in the z-direction, respectively.

## Clinical impact of UHR imaging

The overall impact of the improved spatial resolution on the diagnostic interpretation of cardiothoracic CT scans performed at UHR-PCCT has yet to be fully explored. UHR-PCCT with the smaller detector element size in the fan direction and the small pixel effect allows for superior image quality in ultra-low-dose examinations, with considerable potential for radiation dose reduction [45]. Moreover, the only recent addition of spectral data at UHR could further enhance the power of UHR imaging in cardiothoracic examinations in the near future.

In chest imaging, the improved spatial resolution could empower the characterization of complex lung diseases like interstitial lung diseases (ILD) and combined pulmonary fibrosis and emphysema for instance, by better differentiating and quantifying the disease sub-entities, like subpleural fibrosis and paraseptal emphysema in combined pulmonary fibrosis and emphysema, the different lung patterns in ILDs, including ground-glass, reticulation, honeycombing, and traction bronchiectasis [42]. The improved image quality was coupled with an improved diagnostic confidence in usual interstitial pneumonia diagnosis [46] and in the assessment of ILD progression over time in patients with systemic sclerosis-related ILD [47]. In these studies, the UHR mode with 0.2 mm slice thickness and 1,024 × 1,024 matrix showed the potential to enable early detection of even subtle manifestations of ILD.

**Fig. 5:**
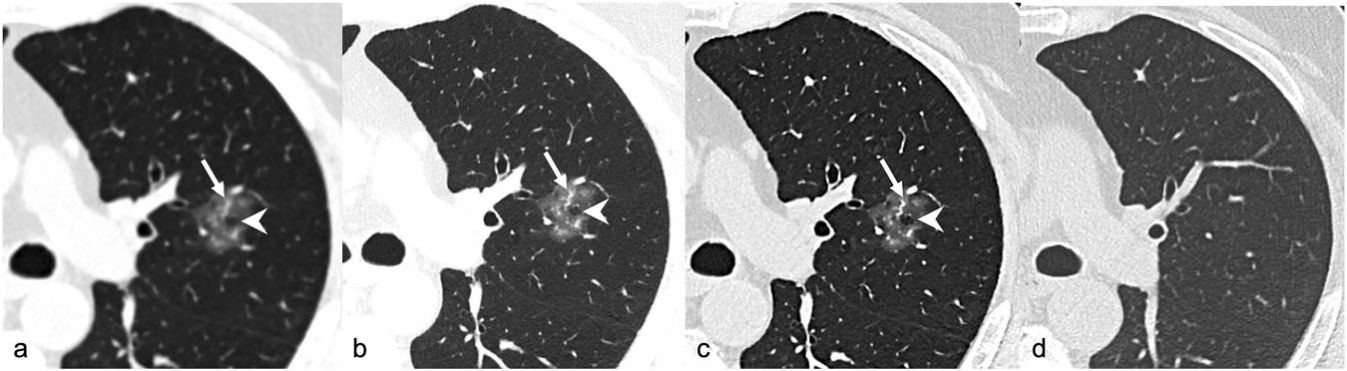
Axial chest PCCT scan of a patient with a pure ground-glass lung nodule in the upper left lobe. The inflammatory nodule shows ill-defined margins, bubble-like lucencies (arrowhead) and a serpiginous dilated vessel (arrow) within it. The characterization of these peculiarities of the nodule improves as matrix size increases (from 512 × 512 (**a**) to 1,024 × 1,024 (**b**, **c**), slice thickness decreases (from 1 mm with 0.6 slice increment in **a** and **b** to 0.4 mm with 0.2 slice thickness in **c**) and a dedicated reconstruction kernel for lung (Bl60) is used (**c**). The inflammatory ground-glass nodule, along with the inner vessel dilation and bubble-like lucencies, completely disappeared at follow-up scan (**d**), acquired by a conventional EID-CT scanner (1.25 mm slice thickness, 0.6 mm increment, matrix size 512 × 512, Br56 reconstruction kernel)

**Fig. 6:**
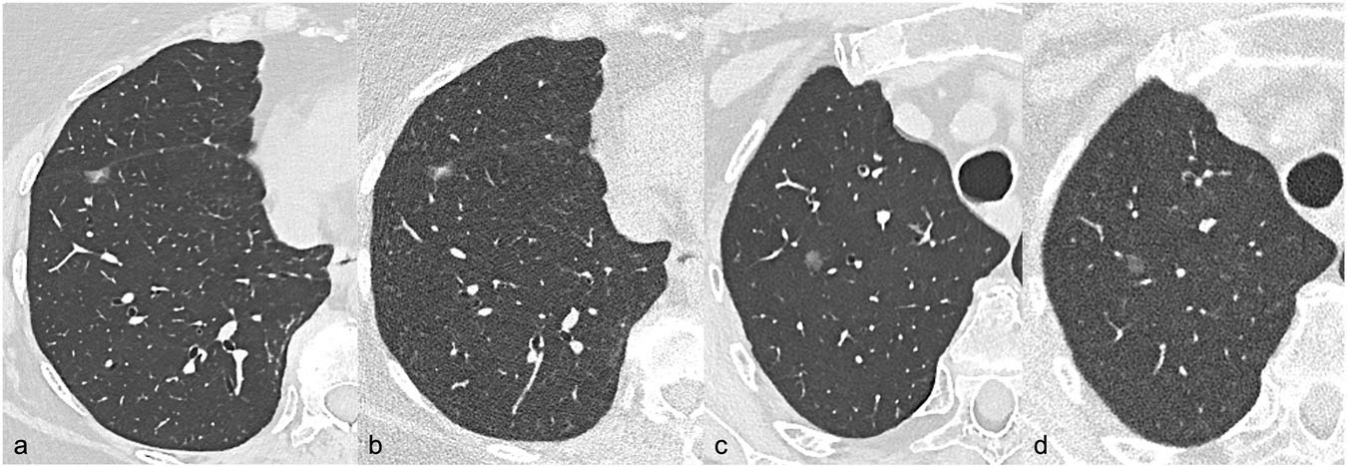
PCCT scans of a part-solid nodule (**a**, **b**) and a pure ground-glass nodule (**c**, **d**) at lung window setting obtained with the same reconstruction parameters (slice thickness/slice increment 0.4/0.2 mm, reconstruction kernel Bl64, Matrix 768 × 768, QiR4) at full dose (**a**, **c**) and ultra-low dose (**b**, **d**) in the same patient. The low-dose images (**b**, **d**) allow the accurate assessment of morphology and density features as well as of the dimensions of the subsolid nodules, including the differentiation and measurement of the solid component (6 mm) of the part-solid nodule. Acquisition protocols (full dose *versus* ultra-low dose): kV 120 *versus* Sn100, IQ level 90 *versus* 8, effective mAs 79 *versus* 55, CTDI vol 6.37 L *versus* 0.47 L, DLP mGycm 212 *versus* 16 (mSv 2.9 *versus* 0.22)

Regarding focal lung diseases, the higher spatial resolution of PCCT enables the correct identification of vessels within subsolid lung nodules (Fig. 5), avoiding their misinterpretation with a solid component (Fig. 6). Wang et al demonstrated the superior characterization of subsolid nodules by using PCCT as compared to same-day EID-CT in 89 lung nodules, also at significantly lower radiation doses (1.8 mSv *versus* 2.2 mSv) [48]. Jungblut et al demonstrated that at half dose, PCCT can depict lung nodules similar to full dose, and at quarter dose, PCCT performs comparably to standard low-dose EID-CT [49]. Indeed, ultra-low-dose chest PCCT is feasible with a reduction of 40 to 90% of radiation dose, without any loss in diagnostic information or in signal-to-noise ratio, as demonstrated both *in vitro* [50] and *in vivo* [51]. Ultra-low-dose PCCT can also accurately depict lung abnormalities in such complex patients as lung transplanted ones, with a tenfold radiation dose reduction (down to 0.26 or 0.17 mSv) [51]. This may result in sustainable thoracic radiology, especially in patients undergoing several follow-up timepoints as those undergoing antifibrotic treatment in ILD.

In cardiovascular studies, an improved spatial resolution in PCCT empowers plaque visualization and characterization (Fig. 3), stenosis quantification, evaluation of in-stent re-stenosis (Fig. 4), patient risk stratification (by coronary artery calcium score—CACS), FFR and microvascular disease assessment. Si-Mohamed et al [35] demonstrated an increase in diagnostic confidence of radiologists and an improved image quality of PCCT *versus* EID-CT for assessing coronary calcifications, stents, and noncalcified plaques of 100%, 92%, and 45%, respectively.

The UHR of PCCT empowers a sustainable radiology in cardiovascular imaging by permitting an accurate diagnostic evaluation of coronaries in patients previously excluded with previous generations of CT scanners, such as patients with high calcium load, metallic stents, obesity or high heart rates [52]. Using invasive coronary angiography (ICA) as the gold standard, PCCT demonstrated greater sensitivity (100%) and specificity (90%) than EID-CT (75% and 50%, respectively) in the quantification of coronary artery stenosis [53]. Indeed, UHR at 0.2 mm slice thickness and 0.1 mm increment with high-resolution kernels and 1,024 matrix size reduces the blooming artifacts of the calcified plaque, increasing in parallel the noncalcified portion [54]. This translates into a recategorization of CAD-RADS grades and therefore into different patient management.

Halfmann et al studied *in vivo* and *in vitro* coronary stenosis, finding a decreasing median percentage in diameter stenosis with increasing spatial resolution for calcified plaques (ranging from 41% in SR to 35% in HR and 27% in UHR), with a reclassification of 54% of patients to a lower CAD-RADS category than that assigned by standard resolution [55]. Simon et al compared the degree of maximal stenosis and the rate of ICA recommendations in patients who underwent CCTA for suspected CAD with PCCT *versus* those who underwent EID-CT, finding that obstructive CAD was more frequently reported in the EID-CT group despite the higher total CACS in the PCCT group and that PCCT reduced the number of subsequent ICA as compared to patients who underwent EID-CT [56]. Recategorization of patients to lower CAD-RADS grades occurs in 38 to 54% of cases, according to the different studies [53, 55, 57, 58].

Unlike this recategorization in CAD-RADS grades, PCCT allows a radiation dose reduction without recategorization in CACS categories [59]. This important advancement has been demonstrated by using different techniques of spectral imaging. Spectral data enables the well-known iodine maps and VNC (virtual non-contrast) reconstructions, which have been available for more than a decade in DECT imaging. However, only the high spatial resolution of PCCT has now enabled to obtain iodine maps that can depict late myocardial enhancement with an accuracy similar to cardiac MR (85% per patient, > 90% per cardiac segment), with a substantial inter-reader agreement (k = 0.7 per patient, 0.63 per segment) [60].

PCCT permits also calcium-free (virtual non calcium—VNCa) and iodine-free (virtual non iodine—VNI) reconstructions, which may eventually replace true non-contrast (TNC) scans with a significant reduction in radiation dose. VNI outperforms the VNC algorithm in CACS and estimation of the CACS category, with a significant underestimation compared with TNC scans though [61, 62]. There is a strong correlation in CACS between TNC and VNI, with only limited agreement though [63]. The use of low-dose PCD-CT with high-pitch scanning could overcome this issue, but this methodology has only been applied *in vitro* yet [64].

Wang et al demonstrated that PCD-CT yields repeatable and accurate CACS across diverse scanning protocols, recommending Sn100kV, 90 kV, and 120 kV using flash mode at IQ20 for clinical applications considering both accuracy and radiation dose [59].

Apart from CACS, spectral data with VNCa algorithm can be used to further enhance CAD stenosis quantification even in patients with very high CACS and very high heart rates [54, 65]. In particular, Zsarnoczay et al found that UHR improves stenosis characterization up to 100 bpm while VNCa up to 80 bpm [52].

Spectral imaging can also count on the use of virtual monoenergetic images (VMI), which can be reconstructed at different energies in kiloelectron Volt (keV), ranging from 40 to 200 keV [66]. VMI at low keV is used to increase CT attenuation of iodinated structures, potentially increasing the contrast-to-noise ratio, and therefore used in vascular studies. VMI at high keV improves CT attenuation stability and reduces beam-hardening artifacts, and therefore can be used in cardiac studies where in-stent re-stenosis should be assessed. Fahrni et al compared UHR-PCCT and DECT VMIs, finding that UHR at 40 keV VMI on PCCT outperformed 40 keV and 70 keV VMI on DECT for assessing coronary artery stenoses and that blooming artifacts did not increase on PCCT even at higher VMI level (40 *versus* 70 keV) [67]. These results were further corroborated by Wolf et al, who studied the effect of nine different VMI energy levels (40–140 keV) in the assessment of coronary artery stenosis [68]. For calcified and mixed plaque, stenosis severity measurements and blooming artifacts decreased at increasing VMI reconstruction levels, with the highest reduction for calcified plaques (for stenosis from 71% at 40 keV to 57% at 140 keV, for blooming from 78% at 40 keV to 49% at 140 keV) [68].

VMI may mitigate inconsistencies in traditional density measurements that result from in-scan and inter-scan related factors [69, 70]. Thus, VMI may become the key for any quantitative imaging study, as it improves the standardization of density numbers.

In line with standardization of density measurements, Salyapongse et al demonstrated that deep silicon PCDs provide HU values closest to ideal numbers and less variable than single-energy and dual-energy EIDs for air, water, iodine and bone and consequently a greater stability and accuracy of CT densities that can facilitate quantitative tasks [71]. Once corroborated by further studies, this observation could be translated into more accurate and reproducible quantitative studies.

Regarding conventional quantitative imaging, Sotoudeh-Paima et al [72] demonstrated a superior qualitative and quantitative performance of PCCT over EID-CT counterparts, with a reduction of about 60% in the error of mean LAA_-950_, the imaging biomarker of emphysema, as stated by the Fleischner Society recommendations [73, 74].

Regarding more advanced quantitative imaging, Chen et al found radiomics signatures with a better discriminative value to identify coronary plaques at risk of rapid progression as compared with conventional morphological plaque parameters [75]. However, radiomics and AI models are hugely affected by data variability among CT scanners, vendors, acquisition protocols and image post-processing techniques, and variability in radiomics features. New techniques based on VMI or deep silicon PCCT may reduce quantification errors in the near future. Thus, further studies aimed at standardizing PCCT imaging acquisition and reconstruction protocols in cardiovascular and lung examinations would be highly invaluable in the next years, first for research and then for clinical practice.

## Challenges of UHR at PCCT

Although UHR-CT imaging offers exceptional spatial detail, it comes with limitations and challenges. All the challenges must be carefully balanced against the diagnostic benefits to ensure effective and safe use of UHR-CT imaging in clinical practice.

One major concern is the vast amount of data generated, which requires advanced processing power, storage capacity, and specialized software, which can be costly and complex to manage. UHR-CT systems also demand higher technical expertise to optimize scan protocols and interpret the resulting numerous images accurately. Another concern regards slower acquisition times. Although UHR-CT imaging can carry slower acquisition times, fast-rotating UHR-capable CT systems (*e.g*., dual-source and PCCT), fast gantry speeds and wide detector arrays can offset the slower acquisition tendency, keeping total scan time short. Optimization of CT protocols is of utmost importance to reduce scanning time, but also to reduce image noise and radiation dose.

Indeed, radiation dose is another important concern of UHR-CT imaging, raising patient safety issues, especially in repeated chest imaging. However, UHR at PCCT often enables dose reduction while improving image quality both in cardiac and chest CT imaging. Ultra-low-dose chest PCCT is feasible with a reduction of 40 to 90% of radiation dose, without any loss in diagnostic information or in signal-to-noise ratio [50, 51]. Moreover, multiple peer-reviewed studies demonstrated that PCCT can reduce radiation dose by 20% up to over 60% in various cardiac imaging applications, including CCTA, stent evaluation, TAVI planning, and CACS [51, 76–78]. The reductions are achieved while maintaining or even improving image quality. Thus, the concern of the increase in dose is a topic of interest for further research, as it could be overcome by the optimization of CT protocols.

## Conclusions

PCCT scanners enable radiologists to evaluate cardiothoracic examinations with an exceptional spatial resolution, along with spectral information and dose reduction. The UHR in cardiothoracic imaging enables cardiovascular assessment in patients previously excluded or in those with non-diagnostic studies, such as obese patients or those with extensive calcifications or metallic stents. Furthermore, UHR empowers the visualization and follow-up of lung patterns of focal and diffuse diseases at unprecedentedly low radiation doses.

## Data Availability

Images included in the manuscript are available at the institution.

## Abbreviations

CACS: Coronary artery calcium score
CT: Computed tomography
DECT: Dual-energy computed tomography
EID: Energy integrating detector
FOV: Field-of-view
HR: High resolution
HU: Hounsfield units
ICA: Invasive coronary angiography
ILD: Interstitial lung disease
keV: Kilo-electron volt
kV: Kilovolt
MPR: Multiplanar reconstructions
MR: Magnetic resonance
MTF: Modulation transfer function
PCCT: Photon-counting computed tomography
PCDs: Photon-counting detectors
QIR: Quantitative iterative reconstructions
TNC: True non-contrast
TTF: Task transfer function
UHR: Ultra-high resolution
VMI: Virtual monoenergetic images
VNC: Virtual non-contrast
VNCa: Virtual non calcium
VNI: Virtual non iodine
